# Assessment of the Suna trap for sampling mosquitoes indoors and outdoors

**DOI:** 10.1186/s12936-019-2680-7

**Published:** 2019-02-22

**Authors:** Monicah M. Mburu, Kennedy Zembere, Alexandra Hiscox, Jomo Banda, Kamija S. Phiri, Henk van den Berg, Themba Mzilahowa, Willem Takken, Robert S. McCann

**Affiliations:** 1 College of Medicine, University of Malawi, Blantyre, Malawi; 2 Laboratory of Entomology, Wageningen University and Research, Wageningen, The Netherlands; 3 MAC Communicable Diseases Action Centre, Blantyre, Malawi

**Keywords:** Anophelines, Culicines, CDC-LT, HLC, Suna trap, Simultaneous use, Sampling, Efficiency, Indoors, Outdoors

## Abstract

**Background:**

Entomological monitoring is important for public health because it provides data on the distribution, abundance and host-seeking behaviour of disease vectors. Various methods for sampling mosquitoes exist, most of which are biased towards, or specifically target, certain portions of a mosquito population. This study assessed the Suna trap, an odour-baited trap for sampling host-seeking mosquitoes both indoors and outdoors.

**Methods:**

Two separate field experiments were conducted in villages in southern Malawi. The efficiency of the Suna trap in sampling mosquitoes was compared to that of the human landing catch (HLC) indoors and outdoors and the Centers for Disease, Control and Prevention Light Trap (CDC-LT) indoors. Potential competition between two Suna traps during simultaneous use of the traps indoors and outdoors was assessed by comparing mosquito catch sizes across three treatments: one trap indoors only; one trap outdoors only; and one trap indoors and one trap outdoors used simultaneously at the same house.

**Results:**

The efficiency of the Suna trap in sampling female anophelines was similar to that of HLC indoors (P = 0.271) and HLC outdoors (P = 0.125), but lower than that of CDC-LT indoors (P = 0.001). Anopheline catch sizes in the Suna trap used alone indoors were similar to indoor Suna trap catch sizes when another Suna trap was simultaneously present outdoors (P = 0.891). Similarly, catch sizes of female anophelines with the Suna trap outdoors were similar to those that were caught outdoors when another Suna trap was simultaneously present indoors (P = 0.731).

**Conclusions:**

The efficiency of the Suna trap in sampling mosquitoes was equivalent to that of the HLC. Whereas the CDC-LT was more efficient in collecting female anophelines indoors, the use of this trap outdoors is limited given the requirement of setting it next to an occupied bed net. As demonstrated in this research, outdoor collections are also essential because they provide data on the relative contribution of outdoor biting to malaria transmission. Therefore, the Suna trap could serve as an alternative to the HLC and the CDC-LT, because it does not require the use of humans as natural baits, allows standardised sampling conditions across sampling points, and can be used outdoors. Furthermore, using two Suna traps simultaneously indoors and outdoors does not interfere with the sampling efficiency of either trap, which would save a considerable amount of time, energy, and resources compared to setting the traps indoors and then outdoors in two consecutive nights.

**Electronic supplementary material:**

The online version of this article (10.1186/s12936-019-2680-7) contains supplementary material, which is available to authorized users.

## Background

Control of adult malaria mosquitoes in Africa has been primarily based on the use of insecticides applied either on the inner walls of houses (indoor residual spraying (IRS)) or by impregnating bed nets. As a result, significant reductions in malaria cases have been achieved [1]. However, there are concerns on the long-term effectiveness of such tools because, since the introduction of these chemicals for malaria control, widespread resistance of anopheline mosquitoes has been reported [2–8]. Furthermore, changes in the biting behaviour of malaria vectors have been reported following the use of long-lasting insecticidal nets (LLINs) [9–12].

When assessing the impact of vector control tools on malaria vector populations, entomological monitoring provides important data on the species composition of mosquito communities, the abundance of each species contributing to malaria transmission in a region, the biting behaviour of these mosquitoes, and the susceptibility of mosquitoes to insecticides. A variety of methods for sampling mosquitoes exist, most of which are biased towards, or specifically target, certain portions of a mosquito population (e.g. host-seeking females or resting mosquitoes). Therefore, it is important to understand the strengths and weaknesses of any sampling method to determine whether it is appropriate for addressing a specific question about the behaviour of malaria vectors [13, 14].

Host-seeking females are considered the most epidemiologically relevant portion of a mosquito population because they are directly responsible for disease transmission through blood feeding [15, 16]. The gold standard for measuring host-seeking malaria mosquitoes (*Anopheles* spp.) has traditionally been the human landing catch (HLC), whereby mosquitoes are captured as they land to feed on a human host [17]. The HLC method directly estimates the peak biting times for vectors, the vectors’ indoor/outdoor biting preferences and the number of infectious bites that a single individual can receive per unit time. One limitation of HLC is that it is labour intensive, requiring collectors to be alert and active throughout each night of the sampling period. Additionally, standardization of HLC across sampling points is restricted by differences among people in their attractiveness to mosquitoes and ability to collect mosquitoes. Concerns about exposing HLC volunteers to malaria during sampling have also been raised, but providing collectors with a prophylactic drug during the sampling period significantly minimizes the risk of malaria infection [18].

Alternatively, mechanical traps targeting host-seeking female *Anopheles* have been developed as potential substitutes to HLC [19]. These include the CDC-LT, which is typically placed next to a person sleeping under a bed net [20], whereby the person acts as an attractive stimulant for mosquitoes [21], but the mosquitoes are not able to reach the person because of the bed net. Mosquitoes are subsequently caught by the fan-driven suction system of the CDC-LT as they fly near the filament bulb lighting the trap, though the benefit of adding light to the trap beyond the attraction of the human host may be limited [21, 22]. While CDC-LT requires less labour than HLC, variation among humans in their attractiveness to mosquitoes [23] still inhibits standardization of the CDC-LT across sampling points. Comparisons of the sampling efficiency of the CDC-LT relative to that of the HLC have given variable results in different regions [20, 24–27], indicating that a more standardized method for sampling host-seeking *Anopheles* is needed. Furthermore, CDC-LT sampling of *Anopheles* is primarily designed for indoor sampling, given the requirement of setting it next to an occupied bed net. When used outdoors, the CDC-LT generally collects very few *Anopheles* [22]. Therefore, a better alternative to HLC than CDC-LT is needed.

Other mechanical traps target host-seeking female *Anopheles* using chemical baits composed of volatiles found on human skin [28–32], which are attractive to host-seeking *Anopheles*. For instance, the Suna trap is an odour-baited trap that has recently been developed to collect host-seeking mosquitoes both indoors and outdoors [33]. To attract mosquitoes, it uses a synthetic blend of chemicals found on human skin [34, 35] and carbon dioxide (CO_2_) produced through a process of yeast and molasses fermentation [36]. The odour blend is standardized, allowing for reliable comparisons among trapping locations. It does not require any human interaction between trap set up in the afternoon and collecting the mosquitoes from the trap the next morning. The minimal labour requirements, the ability for use outdoors, and capacity for standardization make the Suna trap a promising alternative for large-scale monitoring of *Anopheles* populations.

The positioning of traps during entomological monitoring is also important [14]. As the Suna trap can be used for collection of mosquitoes both indoors and outdoors, considerable time could be saved if the trap could be used simultaneously indoors and outdoors. However, there are concerns about possible competition between traps under such arrangements, whereby the presence of one trap may affect the catch of the other trap. Therefore, the objectives of this study were to compare the efficiency of the Suna trap in sampling mosquitoes relative to the HLC and the CDC-LT and to assess the effect of the simultaneous use of the Suna trap indoors and outdoors on the collection of mosquitoes in each trap.

## Methods

### Study site

Two separate field experiments were conducted in rural villages of Chikhwawa District, in southern Malawi. The villages lie along the lower Shire valley and experience a single rainy season from November through April. The main malaria vectors in the region are *Anopheles gambiae* sensu stricto (s.s.), *Anopheles funestus* and *Anopheles arabiensis* [37, 38]. Malaria transmission occurs throughout the year with the rates intensifying during the rainy season. The region is characterized by subsistence farming, and most of the houses have mud or clay-brick walls with grass-thatched or iron-sheet roofs. Each study was conducted in villages that were part of the Majete Malaria Project (MMP), a cluster-randomized malaria control trial which has been described in detail by McCann et al. [39]. The experiment assessing the efficiency of the Suna trap in sampling mosquitoes, took place in two neighbouring villages, namely Tsekera (− 15.985, 34.78) and Chipula (− 15.990, 34.78) in 2014 before the MMP trial activities began. The study on the simultaneous use of the Suna trap indoors and outdoors took place in Chigwata II (− 16.02, 34.52) and Kalonga (− 16.02, 34.51) villages in 2017. These two villages were under the control arm of MMP (i.e. no larval source management or house improvement was implemented in these two villages).

### Comparing the efficiency of the Suna trap in sampling mosquitoes

In Tsekera and Chipula, ten houses representative of the local setting were selected as locations for sampling mosquitoes based on the following criteria: houses with open eaves, grass thatched roofs, mud walls, three to five people sleeping in the house each night, and the residents did not normally cook inside the house or on the veranda. All houses were of a similar size and were at least 50 m from each other.

Mosquitoes were sampled using three methods: the Suna trap, CDC-LT and HLC. Two of these methods (Suna trap and HLC) were used both indoors and outdoors. The CDC-LT was only used for indoor sampling based on previous studies [22]. Thus, there were five treatments in the experimental design: Suna trap indoors or outdoors; HLC indoors or outdoors; and CDC-LT indoors. Mosquitoes were sampled five nights per week for 8 weeks from 7 July to 29 August 2014, except for one night missed due to field supervisor illness, resulting in 39 sampling nights. The five treatments were rotated through ten houses according to a Williams design (Additional file 1) to control for any potential effects of the sequence in which they were used at a house [40].

A solar power system was set up at each house to run the Suna trap and CDC-LT. A solar panel was set on the roof and connected to a controller, battery and timers to run the traps. The timers were set to turn on a trap at 17:00 h on the day it was scheduled to run according to the study design (Additional file 1) and turn off at 07:00 h the following morning. The CDC-LT were suspended 50 cm above the floor in the bedroom, at the foot of a bed where a resident of the house slept under their own insecticide-treated bed net [20]. Suna traps were suspended with the entry 30 cm above ground level [33]. For outdoor sampling, a Suna trap was hung at the side of the house from an overhanging eave. For indoor sampling, a Suna trap was hung in the sitting room. Suna traps were baited with the MB5 blend of attractants [34, 35]. The medium for dispensing the MB5 blend was similar to that of Mweresa et al. [41], which consists of an absorbent layer (95% cellulose and 5% sodium polyacrylate fibres) of a disposable menstrual sanitary pad (unscented Always ultra-thin, ultra-fine Gel-X, Fabricadona Egiptopor, EG Procter & Gamble Company, Egypt). Suna traps were supplied with CO_2_ produced through a process of yeast and molasses fermentation [36] prepared each night of sampling. Household residents were informed about the operation of the traps and instructed not to interfere with the traps or solar power system.

For HLC, eight male collectors were recruited from the study villages. Prior to the study, all collectors were trained in the HLC technique and tested for malaria using a rapid diagnostic test (RDT). Collectors with a positive RDT were treated with artemether–lumefantrine according to current national treatment guidelines. All collectors were given malaria prophylaxis with doxycycline at a daily dose of 100 mg for the duration of exposure (8 weeks) and for 30 days thereafter. A team of two people worked at each house assigned for HLC, working one-after-the-other in two 7-h shifts. The first shift was from 17:00 h to midnight and the second from midnight to 07:00 h. Each hour, collectors worked for 45 min followed by a 15-min break. A field supervisor did sporadic spot checks to ensure HLC collectors were following protocol. For indoor sampling, the collector sat in the sitting room of the house. For outdoor sampling, the collector sat about 1–3 m from the front door of the house. Collectors sat with their legs exposed from their knees down and collected mosquitoes that landed on their legs using a mouth aspirator. Mosquitoes were then gently blown into a paper cup that had been pre-labelled with the house number, date and hour of collection.

### Assessing the simultaneous use of Suna trap indoors and outdoors

Twelve houses that had the following criteria were recruited for the study in Kalonga and Chigwata II villages: open eaves, grass-thatched roofs, houses that were ≥ 25 m apart and at least 100 m away from any mosquito breeding habitat. Placement of Suna traps comprised of: (a) a single trap indoors, (b) a single trap outdoors and (c) two traps (one indoors and one outdoors) at the same house, simultaneously (Additional file 2). Mosquito sampling was carried out four nights per week from 23 March to 19 May 2017 (during the rainy season). To rule out order effects, a 12 × 12 experimental design was adopted (Additional file 3). The set-up of the Suna traps was similar to that described above with the following exceptions: the medium for dispensing the MB5 blend was a manufactured cartridge (BG-MB5 blend dispenser, Biogents, Regensburg, Germany); and the batteries were charged at an MMP research station and moved to the study houses each afternoon rather than using solar power systems to charge the batteries at the houses.

### Identification of mosquitoes and detection of *Plasmodium falciparum* DNA

All mosquitoes were taken to the laboratory for processing. They were identified using the protocol by Gillies and Coetzee [42]. All *Anopheles* were identified as either *An. gambiae* sensu lato (s.l.), *An. funestus* s.l., *Anopheles coustani* or *Anopheles tenebrosus* and the abdominal status was recorded. There was no further classification of culicines beyond the subfamily level. Female *An. gambiae* s.l. and *An. funestus* s.l. were further identified to species level using PCR. The head and thoraces of all female *An. gambiae* s.l. and *An. funestus* s.l. were tested for the presence of *Plasmodium falciparum* DNA using qPCR [43] with a Ct value ≤ 37.0 as the cut-off for *P. falciparum* positive.

### Data analysis

For both studies, generalized linear models with a Poisson distribution and a log link function were used to assess differences among the treatments in the number of female anophelines and culicines collected per night. For each set of analyses, the two outcomes assessed were the number of female anophelines and the number of female culicines caught per house, per night. Generalized estimating equations were used to account for repeated measures by house. The three sampling methods used indoors in the first study (Suna trap, HLC and CDC-LT) were compared in one set of analyses, while the two methods used outdoors (Suna trap and HLC) were compared in a separate set of analyses. For the study assessing whether the simultaneous use of Suna traps in the same house leads to competition between the traps, three comparisons were made: (1) the numbers of mosquitoes collected indoors (without another trap outdoors) were compared to the numbers collected indoors when a trap was used simultaneously outdoors; (2) the numbers of mosquitoes collected outdoors (without another trap indoors) were compared to the numbers collected outdoors when a trap was used simultaneously indoors; and (3) the numbers of mosquitoes collected indoors (combined across treatments) were compared to the numbers of mosquitoes collected outdoors (also combined across treatments). A number of variables were included as covariates in these models: the number of people that slept in the house the previous night, wall type, use of bed net, cooking location and kind of livestock that stayed within 20 m of the house the previous night. Wall type was categorized as mud, fire-baked bricks and sun-dried bricks. Cooking locations were: inside the house, on the veranda, outside the house but within 2 m, and outside more than 2 m from the house. Livestock comprised of cattle, goats, and chickens. Floor and door types were not included as covariates because all the floors and doors were made of mud and wood, respectively. All analyses were performed using IBM SPSS statistics, version 20.0.

## Results

In the experiment comparing the efficiency of the Suna trap in sampling mosquitoes relative to the HLC and the CDC-LT, a total of 2458 mosquitoes were collected. Of these, 8% were female anophelines, 87% female culicines, 1% male anophelines and 4% male culicines. Of the female anophelines, catches comprised of *An. gambiae* s.l. (59%; n = 116) and *An. funestus* s.l. (41%; n = 82). Out of the 198 female anophelines, 189 were analysed molecularly using PCR. Of the 189, 115 were identified as *An. arabiensis*, 50 as *An. funestus* s.s. and 5 as. *Anopheles parensis*. Nineteen of the anophelines could not be identified further because they failed to amplify. Most of the female anophelines were unfed, but some fed, half-gravid or gravid female anophelines were also collected (Table 1).

**Table 1:** Number of mosquitoes collected for the study comparing the efficiency of the Suna trap in sampling mosquitoes

	Unfed	Fed	Half gravid	Gravid	Undetermined	Total females	Male
*An. gambiae* s.l.							
CDC-LT indoors	53	19	6	8	2	88	4
HLC indoors	3	5	0	0	0	8	1
HLC outdoors	6	1	0	0	0	7	0
Suna indoors	3	4	0	0	0	7	0
Suna outdoors	6	0	0	0	0	6	2
*An. funestus* s.l.							
CDC-LT indoors	29	22	3	2	0	56	11
HLC indoors	0	4	0	0	0	4	0
HLC outdoors	3	0	0	0	0	3	0
Suna indoors	6	0	0	0	1	7	1
Suna outdoors	9	2	0	0	1	12	2
Culicines							
CDC-LT indoors	263	18	0	7	0	288	49
HLC indoors	567	26	2	10	0	605	13
HLC outdoors	966	47	3	20	0	1036	16
Suna indoors	49	0	0	0	0	49	4
Suna outdoors	169	2	0	1	0	172	7

Of the 189 female anophelines tested for the presence of *P. falciparum* DNA, 34 (30 *An. arabiensis* and 4 *An. funestus* s.s.) were positive for *P. falciparum* DNA, indicating a sporozoite rate of 18% across sampling methods and locations. Of these, 27 were from the CDC-LT (sporozoite rate of 19%), 4 were from HLC indoors (33%), 2 were from HLC outdoors (20%) and 1 was collected indoors with the Suna trap (7%).

Twenty-one house-nights were excluded from the analysis due to incomplete sampling effort (e.g. dead battery or the owner of the house was unavailable), resulting in 369 total house-nights analysed instead of 390.

**Fig. 1:**
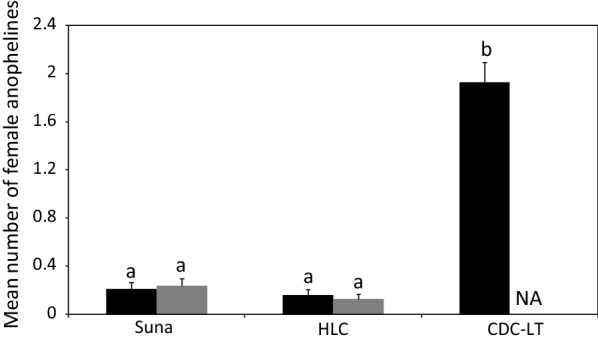
Mean number (± SE) of female anophelines caught indoors (black bar) and outdoors (grey bar) with Suna traps, HLC and CDC-LT. NA indicates the absence of a trap in a different location. Bars with different letters denote differences within location (i.e. indoors/outdoors)

**Fig. 2:**
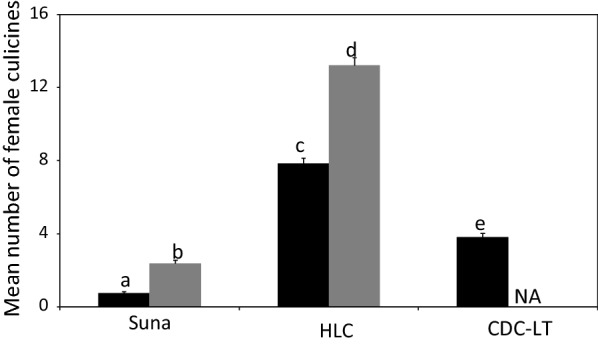
Mean number (± SE) of female culicines caught indoors (black bar) and outdoors (grey bar) with Suna traps, HLC and CDC-LT. NA indicates the absence of a trap in a different location. Bars with different letters denote differences within location (i.e. indoors/outdoors)

Indoors, catches of female anophelines with Suna traps were similar to those of the HLC (risk ratio (RR) = 0.66, 95% confidence interval (CI) 0.32–1.38, P = 0.271) but lower than those of the CDC-LT (RR = 8.18, 95% CI 4.95–13.53, P = 0.001). For outdoor sampling, catches with Suna trap were similar to those of the HLC (RR = 0.54, 95% CI 0.25–1.19, P = 0.125; Fig. 1). For female culicines, indoor collections with the Suna trap were lower than those of the HLC (RR = 3.27, 95% CI 2.76–3.87, P = 0.001) and the CDC-LT (RR = 1.59, 95% CI 1.32–1.92, P = 0.001). Likewise, outdoor collections of female culicines with the Suna trap were lower than those of the HLC (RR = 5.51, 95% CI 4.69–6.47, P = 0.001; Fig. 2).

For the study assessing the simultaneous use of the Suna trap indoors and outdoors, the total number of mosquitoes caught was 328. Of these, 3% were males (n = 10) and 97% were females (n = 318). The male mosquito catches comprised of *An. gambiae* s.l. (n = 3) and culicines (n = 7). The female catches comprised of *An. gambiae* s.l. (40.3%; n = 128), *An. coustani* (0.3%; n = 1), *An. tenebrosus* (0.6%; n = 2) and culicines (58.8%: n = 187). Of the 128 female *An. gambiae* s.l., 117 were identified as *An*. *arabiensis* and 11 as *An. gambiae* s.s. Twenty-seven (all *An. arabiensis*) were positive for *P. falciparum* DNA, indicating a sporozoite rate of 21%. Most of the female anophelines were unfed (n = 124) while the rest were either half gravid (n = 3) or fed (n = 2). Sixteen house-nights were excluded from the analysis due to incomplete sampling effort (e.g. dead battery or the owner of the house was unavailable), resulting in 368 total house-nights analysed instead of 384.

**Fig. 3:**
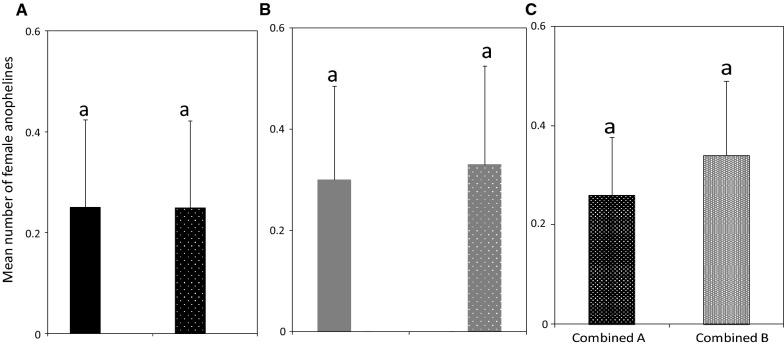
Mean number (± SE) of female anophelines caught with Suna traps. **A** Indoors (black bar), indoors with another trap outdoors (black hatched bar), **B** outdoors (grey bar), outdoors with another trap indoors (grey hatched bar), and **C** combined A and B indicate all female anophelines catches indoors and outdoors, respectively, irrespective of the simultaneous use of trap in either location. Bars with same letters denote the similar number of mosquitoes trapped

Of the total female anophelines, 29 were caught indoors (without another trap outdoors) and 34 were caught outdoors (without another trap indoors). When the indoor and outdoor traps were run simultaneously, the indoor and outdoor catches of female anophelines were 28 and 38, respectively. There were no differences in the number of female anophelines that were caught indoors (without another trap outdoors) and in those that were caught indoors when a trap was used simultaneously outdoors (RR = 1.04, CI 0.61–1.76, P = 0.891; Fig. 3A). Similarly, the number of female anophelines that were caught outdoors (without another trap indoors) were similar to those that were caught outdoors when a trap was used simultaneously indoors (RR = 0.92, CI 0.57–1.48, P = 0.731; Fig. 3B). Pooling across all indoor and outdoor collections irrespective of the simultaneous use of a trap, the catches of female anophelines were similar indoors and outdoors (RR = 0.78, 95% CI 0.55–1.11, P = 0.162; Fig. 3C).

**Fig. 4:**
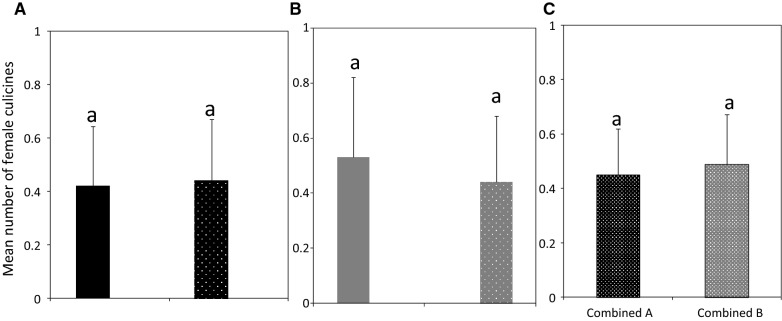
Mean number (± SE) of female culicines caught with Suna traps. **A** Indoors (black bar), indoors with another trap outdoors (black hatched bar), **B** outdoors (grey bar), outdoors with another trap indoors (grey hatched bar) and **C** combined A and B indicate all female culicine catches indoors and outdoors, respectively, irrespective of the simultaneous use of trap in either location. Bars with same letters denote the similar number of mosquitoes trapped

The number of female culicine mosquitoes caught indoors (without another trap outdoors) and outdoors (without another trap indoors) were 44 and 54, respectively. When the indoor and outdoor traps were run simultaneously, the indoor catches were 45 and outdoor catches were 44. There were no differences in the number of female culicine mosquitoes caught indoors (without another trap outdoors) and in those caught indoors when a trap was used simultaneously outdoors (RR = 0.97, CI 0.64–1.48, P = 0.889; Fig. 4A). Likewise, the mosquitoes that were caught outdoors (without another trap indoors) were similar to those that were caught outdoors when a trap was used simultaneously indoors (RR = 1.24, CI 0.83–1.86, P = 0.302; Fig. 4B). Pooling across all indoor and outdoor collections irrespective of simultaneous use of trap, the catches of female culicines were similar (RR = 0.92, 95% CI 0.69–1.23, P = 0.591; Fig. 4C).

Cooking on the veranda was positively associated with female anophelines when the trap was set indoors (without another trap outdoors) and when the trap was set indoors with simultaneous use of another trap outdoors (RR = 3.71, CI 1.42–9.71, P = 0.007). The number of people that slept in the house the previous night was positively associated with the number of female anophelines that were caught outdoors (without another trap indoors) and those that were caught outdoors when a simultaneous trap was used indoors (RR = 1.53, CI 1.12–2.09, P = 0.007). The wall type, rate of bed net usage, presence of cattle, goats, and chickens did not have an effect on the number of female anophelines caught in either location (P ≥ 0.05) (Table 2).

**Table 2:** Effect of covariates on the number of mosquitoes collected for the study assessing the simultaneous use of Suna trap indoors and outdoors

Treatment	Indoors^a^		Outdoors^b^		Combined^c^	
	RR	95% CI	RR	95% CI	RR	95% CI
Female anophelines	1.04	0.61–1.76	0.92	0.57–1.48	0.78	0.55–1.11
People that slept in the house the previous night	1.23	0.87–1.75	1.53	1.12–2.09	1.39	1.10–1.74
Wall type fire baked bricks	0.31	0.04–2.57	1.15	0.41–3.21	0.82	0.34–1.99
Wall type mud bricks	1.59	0.79–3.18	1.47	0.76–2.83	1.52	0.95–2.45
Wall type sun-dried bricks	Ref	–	Ref	–	Ref	–
Mosquito control bed net	0.86	0.43–1.72	1.25	0.69–2.26	1.03	0.66–1.60
Mosquito control none	Ref	–	Ref	–	Ref	–
Cooking inside the house	2.77	0.84–9.17	2.35	0.48–11.43	2.50	0.99–6.31
Cooking on the veranda	3.71	1.42–9.71	1.30	0.63–2.70	2.01	1.14–3.55
Cooking outside, within 2 m of the house	0.83	0.27–2.62	0.91	0.36–2.28	0.88	0.44–1.78
Cooking outside, more than 2 m from the house	Ref	–	Ref	–	Ref	–
Cow	1.21	0.21–6.95	0.14	0.02–1.21	0.40	0.12–1.46
Goat	2.31	0.84–6.36	1.02	0.48–2.20	1.42	0.78–2.57
Chicken	1.07	0.36–3.23	0.38	0.08–1.70	0.70	0.30–1.65
Female culicines	0.97	0.64–1.48	1.24	0.82–1.86	0.92	0.69–1.23
People that slept in the house the previous night	1.18	0.91–1.54	1.02	0.78–1.34	2.0	0.91–1.32
Wall type fire baked bricks	0.51	0.19–1.42	0.43	0.12–1.53	0.49	0.22–1.07
Wall type mud bricks	0.92	0.50–1.67	1.18	0.69–2.02	1.05	0.71–1.57
Wall type sun-dried bricks	Ref	–	Ref	–	Ref	–
Mosquito control-bed-net	0.87	0.51–1.49	0.77	0.48–1.25	0.85	0.59–1.21
Mosquito control-none	Ref	–	Ref	–	Ref	–
Cooking inside the house	0.58	0.15–2.18	0.54	0.16–1.88	0.55	0.23–1.36
Cooking on the veranda	1.0	0.53–1.90	0.60	0.33–1.10	0.75	0.49–1.15
Cooking outside, within 2 m of the house	1.61	0.77–3.38	0.85	0.39–1.88	1.19	0.70–2.03
Cooking outside, away from 2 m of the house	Ref	–	Ref	–	Ref	–
Cow	0.12	0.02–0.96	0.30	0.09–1.09	0.21	0.07–0.61
Goat	0.75	0.38–1.46	0.64	0.36–1.16	0.67	0.43–1.04
Chicken	1.15	0.45–2.92	1.41	0.61–3.23	1.27	0.69–2.34

^a^Description of column A—Suna trap indoors (only) and indoors (with another trap outdoors)

^b^Description of column B—Suna trap outdoors (only) and outdoors (with another trap indoors)

^c^Description of column C—Suna trap indoors and outdoors (with or without another trap indoors or outdoors

There is some evidence that the presence of cattle within 20 m of the house the previous night reduced the catches of female culicines when the trap was set indoors (without another trap outdoors) and when the trap was used indoors with the simultaneous use of a trap outdoors (RR = 0.12 CI 0.02–0.96, P = 0.04). The number of people that slept in the house the previous night, wall type, use of bed nets, presence of goats, and chickens did not have an effect on the number of female culicines caught in either location (P ≥ 0.05) (Table 2).

## Discussion

These studies describe the use of Suna traps for sampling mosquitoes. Comparing the efficiency of the Suna trap relative to the HLC, similar numbers of female anophelines were collected using each method both indoors and outdoors. When assessing whether the simultaneous use of the Suna trap inside and outside a house leads to competition between the two traps, the results demonstrate that the simultaneous use does not affect the catch size in either location. In addition, the observations on the abdominal status showed that most of the female anophelines caught with the Suna trap were unfed, supporting the hypothesis that the Suna trap catches the host-seeking fraction of the anopheline population. Finally, the catch sizes of female anophelines in all indoor collections were similar to those of all outdoor collections in these studies, highlighting the importance of sampling for malaria vectors outdoors in addition to indoors. This sampling can provide data on the relative contribution of indoor and outdoor biting vectors to malaria transmission.

This is the first study of which we are aware comparing the sampling efficiency of the Suna trap with that of HLC, and similar numbers of female anophelines were collected using each method both indoors and outdoors. The Suna trap was designed to mimic a human host, using both CO_2_ and a synthetic odour bait to attract host-seeking mosquitoes. The bait used in the Suna trap was composed of five volatiles normally found on human skin [34, 35], which, when compared to human odour, is equally attractive to female anophelines [29, 34]. While further studies are needed to assess the effect of different environmental conditions on the comparability of the two methods, the results presented here suggest that sampling with the Suna trap can approximate the human biting rate of anophelines in this region.

When compared to the CDC-LT, the Suna trap showed a lower efficiency in sampling both anopheline and culicine mosquitoes. This contrasts with findings from western Kenya where indoor catches with the Suna trap were similar to those of the CDC-LT in a semi-field experiment [33]. One possible explanation could be that the placement of traps relative to sleepers may affect the mosquito catches [29]. Hiscox et al. [33] placed both the Suna trap and CDC-LT next to a person sleeping under a bed-net in a single-room house constructed within a screen-house. In southern Malawi, where the present study was conducted, houses are typically divided into at least two rooms (a bedroom and a sitting room), and the CDC-LT was set in the bedroom next to a person sleeping under a bed-net, following the standard for this sampling method in Africa [20].

The indoor Suna trap sampling, however, took place in the sitting room to match the standard protocol of the HLC method. Differences in the concentrations of odours provided by human hosts between the bedroom and sitting room could have attracted more mosquitoes to the former, where the CDC-LT was located. Further studies on the placement of the Suna trap relative to sleepers are needed. Secondly, differences in sampling efficiencies could be explained by differences in the mosquito species being observed. In their semi-field comparison of the Suna trap and CDC-LT, Hiscox et al. [33] used laboratory reared *An. gambiae* s.s. while in the present study, the most abundant species were *An. arabiensis* and *An. funestus*. It is possible that these two species respond differently to the CDC-LT and/or the Suna trap. Thirdly, the two studies used different media for dispensing the odour baits from the Suna trap. Hiscox et al. [33] used nylon strips [44], and the present study used a sanitary pad absorbent layer [41]. However, this third explanation is unlikely, given that Mweresa et al. [41] collected more anophelines in odour-baited traps using the sanitary pad absorbent layer than nylon strips.

When compared with the HLC indoors, the CDC-LT was more effective in collecting female anophelines in this study, which is in line with findings from Tanzania [45], Kenya [26], and Zambia [46]. However, most studies demonstrate that the two methods collect similar numbers of anophelines [20, 47–50], while others report that the efficiency of the HLC in sampling host-seeking anophelines is higher than that of the CDC-LT [27, 49, 51]. A comprehensive review looking at paired mosquito collections of the HLC and the CDC-LT found that the sampling efficiencies of the two methods vary, in that the CDC-LT catches are either similar to those of the HLC, or they are higher or lower than those of the HLC [52]. Therefore, it is possible that the local environmental conditions affect the efficiency of both sampling methods and may explain the observed differences in catch size.

The HLC and CDC-LT both collected more female culicines than the Suna trap. Moreover, the culicine catch sizes of the HLC were higher than those of the CDC-LT, which is consistent with a study from Zambia [50], but in contrast with that of Mweresa [53], who suggested that the CDC-LT caught more culicines because of the presence of multimodal stimuli (human bait + light). As with anophelines, it is likely that the efficiency of these two methods varies with local mosquito species and environmental conditions.

Combined across the two experiments presented here, the infection rate of *An. arabiensis* with *P. falciparum* was higher than that of *An. funestus.* Though *An. arabiensis* is often seen as a less efficient vector, the abundance of this species, together with its relatively high infection rate, confirms that this species is important as a malaria vector and that it contributes significantly to transmission of malaria in southern Malawi. The absence of *An. funestus* in one of the two studies presented here is most likely explained by seasonal fluctuations. The assessment of simultaneous Suna trap use indoors and outdoors was conducted during the rainy season when densities of *An. arabiensis* are generally higher in this region, while those of *An. funestus* tend to increase at the end of the rainy season and at the beginning of the dry season [38, 54]. Recent findings have shown that this species is still abundantly present in the region [55]. In addition to *An. arabiensis* and *An. funestus, An. gambiae* s.s. was previously common in this region [37, 38], but the current study and others [55] have found very few *An. gambiae* s.s. relative to other anopheline species. The apparent decline of *An. gambiae* s.s. in southern Malawi warrants further investigation, as similar declines in East Africa have been associated with the long-term use of bed nets [56].

In assessing the simultaneous use of the Suna trap indoors and outdoors, the results demonstrate that the trap can be used simultaneously in both locations without any competition. This would save a considerable amount of time, energy and resources when monitoring the abundance of malaria vectors indoors and outdoors, compared to using the trap indoors only and then outdoors only, for 2 consecutive days. Furthermore, the catch sizes of female anophelines collected indoors (with or without the simultaneous use of the trap outdoors) were similar to those that were collected outdoors (with or without the simultaneous use of the trap indoors). This can be explained by the predominance of *An. arabiensis* during this study, as the species exhibits both indoor and outdoor host-seeking behaviour [57]. While indoor mosquito collections are important for assessing vector control programmes, outdoor collections are also essential, in particular with the potential shift towards outdoor biting in some anopheline populations [9, 10, 58]. Therefore, methods for assessing outdoor host-seeking mosquito densities relative to indoor host-seeking mosquito densities are required. The Suna trap addresses this need as a method that provides equal sampling conditions both indoors and outdoors. The Suna trap also requires less labour than the HLC or CDC-LT because it does not rely on the use of humans as baits. The use of a standard synthetic bait in the Suna trap also provides equal sampling conditions across sampling locations, unlike the HLC and CDC-LT, which are subject to differences in attractiveness to mosquitoes among human volunteers [23].

## Conclusion

The efficiency of the Suna trap in sampling host-seeking anopheline mosquitoes was equivalent to that of the HLC. Whereas the CDC-LT was more efficient in collecting female anophelines indoors, the use of the CDC-LT outdoors is limited given the requirement of setting it next to an occupied bed net. As demonstrated in this study, outdoor collections are also essential because they provide data on the relative contribution of outdoor biting to malaria transmission. Therefore, the Suna trap can serve as a better alternative to the HLC and CDC-LT because it does not require the use of humans as natural baits, allows equal sampling conditions across sampling points, and can be used outdoors. Furthermore, using two Suna traps simultaneously indoors and outdoors does not interfere with the sampling of either trap, which saves a considerable amount of time, energy, and resources compared to setting the traps indoors and then outdoors across two consecutive nights.

## Additional files

**Additional file 1.** Nightly schedule of sampling methods used at each house, repeated for eight weeks.

**Additional file 2.** Schematic drawing of Suna trap placement for the study on the simultaneous use of traps.

**Additional file 3.** Nightly schedule of Suna trap placement at each house, for six weeks.

## Abbreviations

HLC: human landing catch
CDC-LT: Centers for Disease Control and Prevention Light Trap
LLINs: long-lasting insecticidal nets
IRS: indoor residual spraying
PCR: polymerase chain reaction
RDT: rapid diagnostic test
MMP: Majete Malaria Project
RR: risk ratio
CI: confidence interval
