# Supplementary material for: Assessment of the Suna trap for sampling mosquitoes indoors and outdoors
Source: Malar J. 2019 Feb 22;18:51. doi: 10.1186/s12936-019-2680-7 (PMC6387520; doi:10.1186/s12936-019-2680-7)
Supplement: Supplementary file 1 — Additional file 1. Nightly schedule of sampling methods used at each house, repeated for eight weeks. [file 12936_2019_2680_MOESM1_ESM.docx]

| House | Sunday | Monday | Tuesday | Wednesday | Thursday |
| --- | --- | --- | --- | --- | --- |
| 1 | CDC-LT (In) | Suna (In) | HLC (In) | Suna (Out) | HLC (Out) |
| 2 | Suna (In) | Suna (Out) | CDC-LT (In) | HLC (Out) | HLC (In) |
| 3 | Suna (Out) | HLC (Out) | Suna (In) | HLC (In) | CDC-LT (In) |
| 4 | HLC (Out) | HLC (In) | Suna (Out) | CDC-LT (In) | Suna (In) |
| 5 | HLC (In) | CDC-LT (In) | HLC (Out) | Suna (In) | Suna (Out) |
| 6 | HLC (Out) | Suna (Out) | HLC (In) | Suna (In) | CDC-LT (In) |
| 7 | HLC (In) | HLC (Out) | CDC-LT (In) | Suna (Out) | Suna (In) |
| 8 | CDC-LT (In) | HLC (In) | Suna (In) | HLC (Out) | Suna (Out) |
| 9 | Suna (In) | CDC-LT (In) | Suna (Out) | HLC (In) | HLC (Out) |
| 10 | Suna (Out) | Suna (In) | HLC (Out) | CDC-LT (In) | HLC (In) |
