# Supplementary material for: Assessment of the Suna trap for sampling mosquitoes indoors and outdoors
Source: Malar J. 2019 Feb 22;18:51. doi: 10.1186/s12936-019-2680-7 (PMC6387520; doi:10.1186/s12936-019-2680-7)
Supplement: Supplementary file 2 [file 12936_2019_2680_MOESM2_ESM.docx]

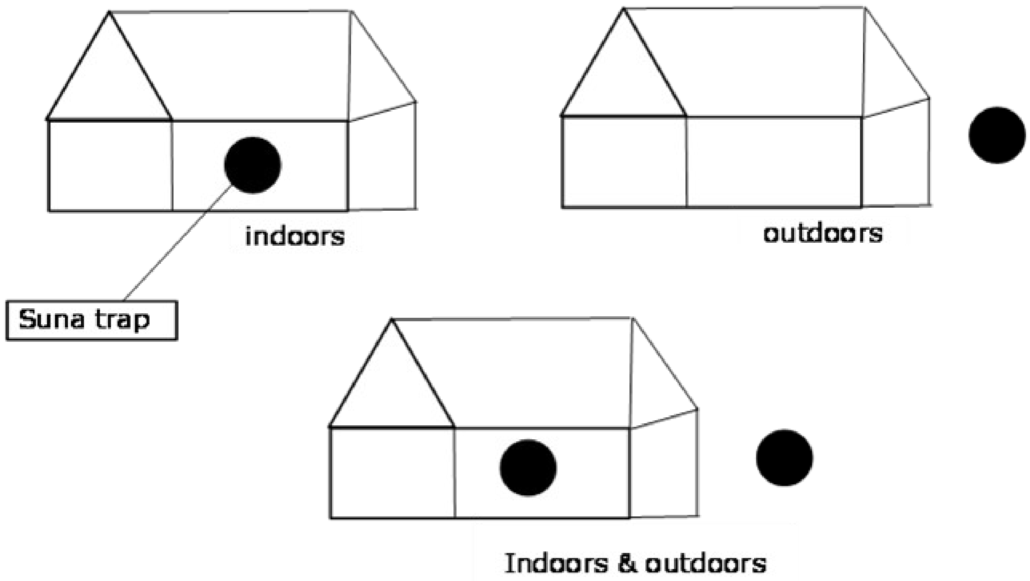
indoors
outdoors
Suna trap
Indoors & outdoors
