# Supplementary material for: Assessment of the Suna trap for sampling mosquitoes indoors and outdoors
Source: Malar J. 2019 Feb 22;18:51. doi: 10.1186/s12936-019-2680-7 (PMC6387520; doi:10.1186/s12936-019-2680-7)
Supplement: Supplementary file 3 [file 12936_2019_2680_MOESM3_ESM.docx]

	Days											
	1	2	3	4	5	6	7	8	9	10	11	12
**Hse**	13	14	15	16	17	18	19	20	21	22	23	24
**1**	in	out	in/out	in/out	in	out	out	in/out	in	in	out	in/out
**2**	out	in/out	out	in/out	in/out	in	out	in	in/out	in	out	in
**3**	in/out	out	in	out	out	in/out	in	in/out	out	in/out	in	in
**4**	in/out	out	in	in	in/out	in	in/out	out	in	out	in/out	out
**5**	in	in/out	out	out	in	out	in	in/out	in/out	in	out	in/out
**6**	out	in/out	out	in	out	in/out	in/out	out	in	in/out	in	in
**7**	out	in	in/out	out	out	in/out	in	in	in/out	in/out	in	out
**8**	in/out	in	in/out	in	in/out	in	out	in	out	in/out	out	out
**9**	in	in	out	in/out	in/out	out	out	out	in/out	in	in	in/out
**10**	in	in/out	in	out	in	in	in/out	out	out	out	in/out	in/out
**11**	out	out	in	in/out	in	out	in/out	in/out	in	out	in/out	in
**12**	in/out	in	in/out	in	out	in/out	in	in	out	out	in/out	out
